# Temporal Development of Dyslipidemia and Nonalcoholic Fatty Liver Disease (NAFLD) in Syrian Hamsters Fed a High-Fat, High-Fructose, High-Cholesterol Diet

**DOI:** 10.3390/nu13020604

**Published:** 2021-02-12

**Authors:** Victoria Svop Jensen, Christian Fledelius, Erik Max Wulff, Jens Lykkesfeldt, Henning Hvid

**Affiliations:** 1 Department of Veterinary and Animal Sciences, Faculty of Health and Medical Sciences, University of Copenhagen, Ridebanevej 9, DK-1870 Frederiksberg, Denmark; jopl@sund.ku.dk; 2 Diabetes Pharmacology 1, Novo Nordisk A/S, Novo Nordisk Park 1, DK-2760 Måløv, Denmark; cfle@novonordisk.com; 3 Gubra ApS, Hørsholm Kongevej 11B, DK-2970 Hørsholm, Denmark; emw@gubra.dk; 4 Pathology & Imaging, Novo Nordisk A/S, Novo Nordisk Park 1, DK-2760 Måløv, Denmark; hhvd@novonordisk.com

**Keywords:** dyslipidemia, nonalcoholic fatty liver disease, nonalcoholic steatohepatitis, animal models, hamster

## Abstract

The use of translationally relevant animal models is essential, also within the field of nonalcoholic fatty liver disease (NAFLD) and nonalcoholic steatohepatitis (NASH). Compared to frequently used mouse and rat models, the hamster may provide a higher degree of physiological similarity to humans in terms of lipid profile and lipoprotein metabolism. However, the effects in hamsters after long-term exposure to a NASH diet are not known. Male Syrian hamsters were fed either a high-fat, high-fructose, high-cholesterol diet (NASH diet) or control diets for up to 12 months. Plasma parameters were assessed at two weeks, one, four, eight and 12 months and liver histopathology and biochemistry was characterized after four, eight and 12 months on the experimental diets. After two weeks, hamsters on NASH diet had developed marked dyslipidemia, which persisted for the remainder of the study. Hepatic steatosis was present in NASH-fed hamsters after four months, and hepatic stellate cell activation and fibrosis was observed within four to eight months, respectively, in agreement with progression towards NASH. In summary, we demonstrate that hamsters rapidly develop dyslipidemia when fed a high-fat, high-fructose, high-cholesterol diet. Moreover, within four to eight months, the NASH-diet induced hepatic changes with resemblance to human NAFLD.

## 1. Introduction

The global obesity and diabetes pandemic is accompanied by a concurrent increase in the prevalence of nonalcoholic fatty liver disease (NAFLD) [1]. This is not surprising, as the liver plays a key role in regulating both fat and glucose metabolism and is intricately involved in processes contributing to the metabolic syndrome [2]. NAFLD is a temporally dynamic liver disease spanning multiple disease stages of increasing histopathological severity and is estimated to affect ~25% of the general population [1]. The initial stage of the disease (termed nonalcoholic fatty liver; NAFL) most often manifests as hepatic steatosis (>5% of hepatocytes with fat infiltration) with no additional hepatocellular injury [3]. A significant fraction (~20–40%) of patients with hepatic steatosis may progress to nonalcoholic steatohepatitis (NASH) within a time-span of 3–6 years [4,5], a pathological stage additionally characterized by hepatic inflammation, hepatocellular damage (ballooning) and in some cases fibrosis [3]. As no pharmacological treatment for NASH and fibrosis is currently available, experimental testing of potential drug candidates in animal models is of major importance. Mice and rats are the most frequently used rodent species in experimental research of NAFLD and NASH [6], and while mouse and rat models have contributed significantly to current knowledge of the disease, important differences in lipoprotein metabolism and plasma lipid profiles exist between these species and humans [7,8,9].

The Syrian hamster has historically been used in research involving other metabolic diseases such as cardiovascular disease due to several similarities of the Syrian hamster’s lipid and lipoprotein metabolism compared to that of humans [10,11]. Although the lipoprotein profile of hamsters is not entirely similar to that of humans, they do carry a considerably larger proportion of their plasma cholesterol in low-density lipoprotein (LDL)-particles compared to both mice and rats [7,12] and similar to humans they express cholesteryl ester transfer protein (CETP)- activity [13], a key enzyme involved in the transfer of cholesterol and triglyceride (TG) between lipoproteins. This becomes relevant in the context of experimental modelling, as lipoprotein metabolism is often altered in human NAFLD [14]. The hamster could as such represent a relevant model for NAFLD research, with higher translational value than mouse and rat models. However, hamster models are currently underrepresented in experimental NAFLD research, and the temporal development of liver pathology in this species after long-term exposure (>25 weeks) to a high-fat, high-fructose diet with added cholesterol is to our knowledge not known.

The aim of this study was therefore to characterize the effects of a putative NASH-inducing diet, with a high content of fat, fructose and cholesterol on metabolic and liver function in Syrian hamsters. The hypothesis was that dietary intake of the NASH-diet would result in marked dyslipidemia and hepatic changes resembling those seen in human NAFLD/NASH. The effects of the NASH-diet were explored during study periods of four, eight and 12 months and compared to two different control diets: A commercial non-purified grain-based chow diet and a semi-purified control diet (designed specifically to match the NASH-diet). This was done to evaluate whether or not the type of control diet (i.e., non-purified vs. semi-purified) influenced the interpretation of the effects of the NASH diet on metabolic and especially hepatic parameters in the study. Marked differences between effects of chow and semi-purified control diets on gut health and adiposity have been shown previously in mice [15]. In the present study we demonstrate that the NASH-diet rapidly induces dyslipidemia in Syrian hamsters and result in development of NAFLD with hepatic alterations resembling changes seen in human patients with NAFLD.

## 2. Materials and Methods

### 2.1. Animals

Ninety male Syrian hamsters (six or seven weeks old) were purchased from Janvier Labs (Le Genest-Saint-Isle, France). Upon arrival, hamsters were housed four per cage and allowed to acclimatize for two weeks before initiation of the study (a hamster aged eight-nine weeks corresponds to a human age of approximately 15 years [16]). During the acclimatization period, hamsters had ad libitum access to a grain-based rodent chow (1324 Altromin, Brogaarden, Denmark) as well as non-chlorinated, non-acidified tap water. Ambient temperature in the housing facility was kept between 20–25 °C, with frequent air changes (8–15 times/h). The relative humidity in the room was between 30–70% and a 12/12 h light/dark cycle was maintained throughout the study. The study was approved by the Danish Animal Experiments Inspectorate in accordance with European Union Directive 2010/63/EU.

### 2.2. Experimental Design

**Figure 1:**
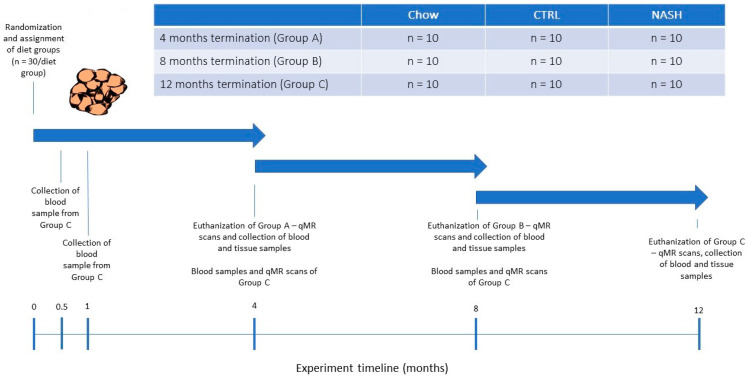
Study Design. At experiment start, male Syrian hamsters (eight to nine weeks old) were randomized to receive either chow diet (Altromin, 1324, Brogaarden), the CTRL-diet (D16010104, Research Diets) or the NASH-diet (D16010102, Research Diets) for four, eight or 12 months (See Group A, B and C in Figure 1). At each time point (four, eight and 12 months), one group/diet group was qMR-scanned, sampled for blood and then euthanized where after tissue samples were collected. Group C (12 months on diet) was additionally sampled for blood at 0.5 and 1 month, to monitor early diet-induced changes in plasma parameters related to dyslipidemia and inflammation/oxidation. qMR: quantitative magnetic resonance.

After the acclimatization period, the hamsters were weighed and then block-randomized by body weight into nine separate groups (n = 10/group, Figure 1) to receive either a grain-based rodent chow (“chow”, 1324 Altromin, Brogaarden, Denmark), a semi-purified low-fat control diet (“CTRL”, D16010104, Research Diets, NJ, USA) or a high-fat high-fructose/cholesterol-enriched diet (“NASH”, D16010102, Research Diets, NJ, USA) for four, eight or 12 months. The contents of the diet formulations are shown in Table 1. More detailed diet formulations can be viewed in Supplemental Table S1. The long study duration was chosen to characterize the effects of long-term exposure to a NASH-inducing diet. Animals were additionally euthanized at four and eight months to monitor the progression of pathological changes in the liver. During the study period, body weight was recorded in all groups on a weekly basis. At four, eight and 12 months, hamsters were scanned using quantitative magnetic resonance (qMR, whole-body scan) to monitor changes in fat mass and lean body mass. In addition, at each time point where hamsters were euthanized plasma and liver samples were collected for biochemical and histological analysis. On the day prior to scheduled euthanasia, the hamsters were fasted for three hours after which blood samples were collected. On the day of euthanasia, the hamsters were weighed and subsequently euthanized by exsanguination while in deep isoflurane anaesthesia. Immediately following euthanasia, the liver was excised and weighed, and liver samples from the left lateral, the right medial and the caudal lobe were collected and transferred to 10% Neutral buffered formalin (Hounisen Laboratorieudstyr A/S, Skanderborg, Denmark) for later histological analysis. Furthermore, six punch biopsies (diameter: 4 mm) were collected from the left lateral lobe and snap-frozen in liquid nitrogen for biochemical analysis. In order to closely follow the development of dyslipidemia, blood samples were collected from the hamsters assigned to the longest study period (12 months) at two weeks and at one month after study initiation. For the remainder of the study blood samples were collected from 12-months hamsters at the same frequency as the hamsters assigned to the other two time points (i.e., at four and eight months).

### 2.3. Quantitative Magnetic Resonance

To follow changes in body composition (estimates of fat and lean tissue mass) all hamsters underwent qMR-scans at four, eight and 12 months using an EchoMRI Body Composition Analyzer (EchoMRI, Houston, TX, USA) according to the manufacturer’s instructions and as previously described [17].

### 2.4. Plasma Parameters

Fasted blood samples (collected after three hours of fasting) were taken from the sublingual vein and transferred to K_3_-EDTA microvette tubes (Sarstedt AG & Co, Nümbrecht, Germany). The samples were centrifuged, plasma was isolated and then kept at −20 °C for later analysis of glucose, free fatty acids (FFA), TG, total cholesterol, low-density lipoprotein cholesterol (LDL-C) and high-density lipoprotein cholesterol (HDL-C), haptoglobin, alanine aminotransferase (ALT), aspartate aminotransferase (AST) and 3-hydroxy-butyrate. Plasma concentrations of all above-mentioned parameters were measured using a Cobas 6000 c501 instrument (Roche Diagnostics GmbH, 68206 Mannheim, Germany) according to the manufacturer’s instructions. In addition, non-HDL-cholesterol fractions were calculated by subtracting the measured HDL-C concentrations from the total cholesterol concentrations. Plasma concentration of hamster insulin was measured using an assay designed for detection of rat insulin as described previously [18]. Throughout the paper, insulin concentrations are therefore given as “rat insulin immunoactivity equivalents” (RIIE) as the exact cross-reactivity to hamster insulin was not determined. Based on the fasting plasma glucose and insulin measurements, homeostatic model assessment of insulin resistance (HOMA-IR)-indexes were calculated using the formula: (fasting plasma glucose (mmol/L) × fasting insulin (µIU/L))/22.5) to assess development of insulin resistance in the hamsters.

### 2.5. Liver Biochemistry

Hepatic concentrations of TG, cholesterol and glycogen were assessed in punch biopsies collected from the left lateral lobe of the liver. In brief, tissue samples were homogenized using a 0.15 M sodium acetate buffer (pH = 4.9) containing 0.75% Triton-X100 (Sigma-Aldrich, Soeborg, Denmark). The homogenized samples were then placed on a heating block (90–100 °C) for two minutes and subsequently cooled on ice. The homogenates produced from each sample were then split into two aliquots. The first aliquot was treated with amyloglucosidase (Sigma Aldrich) to enable the breakdown of glycogen to glucose, and subsequently placed on a heating block (50 °C) for two hours. Then both aliquots were centrifuged at 9000 G for 10 min, and the resultant supernatants were analyzed using a Cobas 6000 c501 instrument (Roche Diagnostics GmbH, Mannheim, Germany) according to the manufacturer’s instructions.

### 2.6. Liver Histology

After fixation in formalin for 48–96 h and subsequent embedding in paraffin, 3 µm sections of liver tissue from the left lateral, the right medial and the caudate lobe were cut and stained with Haematoxylin and Eosin (H&E, Sigma-Aldrich, Soeborg, Denmark) and Picro Sirius Red (PSR, Sigma-Aldrich) for evaluation of hepatic steatosis and collagen deposition, respectively. Additional immunohistochemistry (IHC) was performed for CD68 to characterize development of inflammation and for α-smooth muscle actin (α-SMA) to characterize activation of hepatic stellate cells. IHC for CD68 and α-SMA was performed according to protocols shown in Supplemental Table S2. After staining, all sections were scanned using a NanoZoomer 2.0 HT slide scanner (Hamamatsu, Hamamatsu City, Japan) and subsequently evaluated using NanoZoomer Digital Pathology Image Software (Hamamatsu). Threshold-based image analysis was performed using VIS software (Visiopharm, Hoersholm, Denmark), which quantified the fractional area for each animal with positive staining for each marker of interest (i.e., PSR, CD68, and α-SMA). These area fractions were expressed as the percentage of the total area of liver tissue per animal.

### 2.7. Statistics

All statistical analysis and graphical layout of primary endpoints (defined as parameters measured in liver and plasma samples) and secondary endpoints (all remaining parameters) were done using GraphPad Prism version 8.02 (GraphPad Software Inc., La Jolla, CA, US). Significant differences between groups were tested using the statistical tests described below, at each individual time point. Distribution of the data and variance homology was evaluated by visual inspection of qq-plots and plots of residuals vs. predicted values, respectively. Normally distributed data with homogenous variance were analyzed by one-way ANOVA, followed by pairwise comparisons of groups in *t*-tests with Tukey’s corrections for multiple parallel comparisons. Correction of analyses for multiple testing across biomarkers was not performed. Data with non-normal distribution or heterogenous variance were transformed using the natural logarithm, re-assessed and then analyzed as above. If transformation of the data was not sufficient to meet the assumptions of the ANOVA, the non-parametric Kruskal-Wallis test was applied, followed by Dunn’s test. Data in graphs are presented as group means ± standard error of the mean (SEM). In tables data are presented as mean ± standard deviation (SD). During the study, three animals were euthanized. Two animals due to incidental subcutaneous abscess formation, and one due to excessive weight loss (one from the chow group, one from the CTRL group and one from the NASH group, all belonging to the eight-month time point). During histology image analyses, data from one animal from the chow group at the eight-month time point were excluded (due to abnormal inflammatory infiltration of the liver, which was identified as an outlier upon statistical analysis of the image analysis output). A *p*-value < 0.05 was considered statistically significant.

## 3. Results

### 3.1. The NASH Diet Did Not Induce Hyperglycemia/Hyperinsulinemia But Affected Body Composition

**Figure 2:**
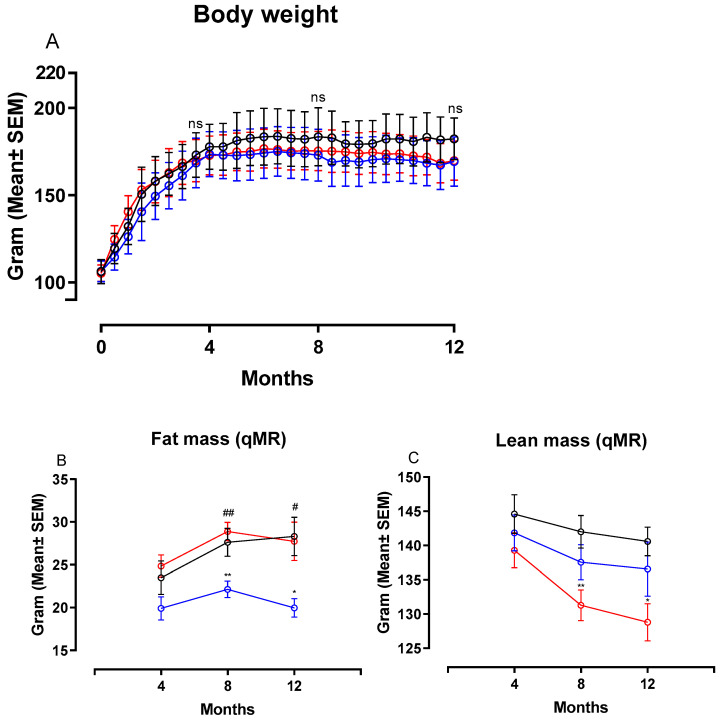
Body weight and body composition after exposure to the NASH and the two control diets. Development in (**A**) body weight, (**B**) fat mass and (**C**) lean mass during the course of the study. Black circles and lines indicate data from chow-fed hamsters. Blue circles and lines indicate data from CTRL-fed hamsters. Red circles and lines indicate data from NASH-fed hamsters. Body weight mean in the three groups are based on group sizes of *n* = 30 from study initiation to four months, *n* = 20 from four to eight months and *n* = 10 from eight to 12 months. Fat and lean mass mean values are based on *n* = 20 at the four- and eight-month time point and *n* = 10 at the 12-month time point. * and ** indicate *p* < 0.05 and *p* < 0.01 compared to chow. # and ## indicate *p* < 0.05 and *p* < 0.01 compared to CTRL. qMR: Quantitative magnetic resonance.

Figure 2 shows changes in body weight (BW), fat and lean mass in hamsters on chow, CTRL and NASH diet during the course of the study. Feeding the NASH diet did not result in statistically significantly different body weights compared to the two control groups at four, eight or 12 months (Figure 2A). Total fat mass was significantly increased in NASH-fed hamsters at eight (*p <* 0.01) and 12 months (*p <* 0.05) compared to the CTRL-fed hamsters, whereas there were no differences between NASH and chow-fed animals (Figure 2B). However, the NASH group had significantly lower lean body mass at eight (*p <* 0.01) and 12 months (*p <* 0.05) compared to the chow group, but not the CTRL group (Figure 2C). Plasma glucose was similar between groups throughout the study, except for the 12-month time point, where a small but significant increase was seen in CTRL hamsters compared to both NASH and chow hamsters (*p <* 0.05, Table 2). Furthermore, plasma concentrations of insulin and calculated HOMA-IR indexes were not significantly different between the groups at any time point (Table 2).

### 3.2. The NASH Diet Induced Marked Dyslipidemia in Syrian Hamsters

**Figure 3:**
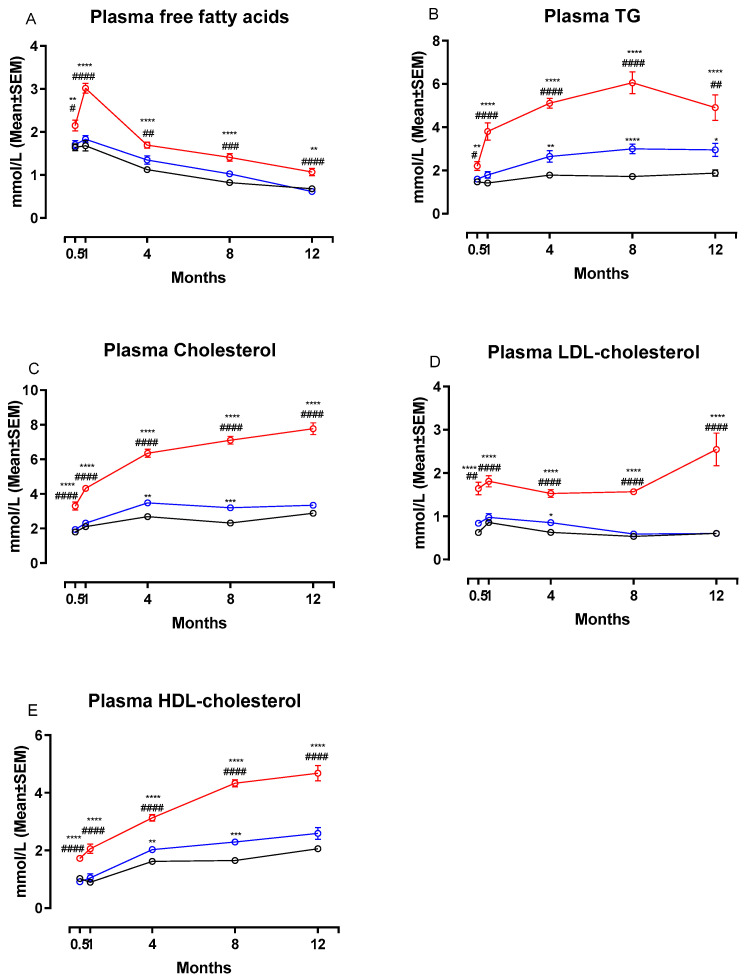
Effects of the NASH diet on lipid parameters in Syrian hamsters fed either NASH, or one of the two control diets during the study period. Effects of the NASH-diet on plasma concentrations of FFA (**A**), triglycerides (**B**) and cholesterol in different lipoprotein fractions (**C**–**E**) during the study period. Black circles and lines indicate data from chow-fed hamsters. Blue circles and lines indicate data from CTRL-fed hamsters. Red circles and lines indicate data from NASH-fed hamsters. Superscripts *, **, ***, **** indicate *p* < 0.05, *p* < 0.01, *p* < 0.001, *p* < 0.0001 compared to chow. Superscripts #, ##, ###, #### indicate *p* < 0.05, *p* < 0.01, *p* < 0.001, *p* < 0.0001 compared to CTRL. FFA: free fatty acid, TG: triglyceride, LDL: Low-density-lipoprotein, HDL: High-density lipoprotein.

Figure 3 shows changes in plasma concentrations of FFA, TG, total Cholesterol, LDL-C and HDL-C during the study. Calculated changes in non-HDL-C are shown in Table 2. The NASH diet induced marked dyslipidemia characterized by significant increases in plasma concentrations of FFA (Figure 3A), TG (Figure 3B), total cholesterol (Figure 3C), LDL-C (Figure 3D) and HDL-C (Figure 3E) compared to both chow and CTRL groups after only two weeks on the diet (*p* < 0.05 or below). Concentrations of all plasma markers remained significantly increased in the NASH group for the remainder of the study. In addition, plasma non-HDL-cholesterol concentrations were significantly increased in NASH-fed hamsters compared to CTRL and chow at all time points (*p <* 0.0001, Table 2). There were also differences between the effects of the two control diets. At four, eight- and 12-months CTRL-fed hamsters had significantly higher plasma concentrations of TG compared to chow (*p* < 0.01, *p <* 0.0001 and *p* < 0.05, respectively, Figure 3B) and at four and eight months higher HDL-C (*p <* 0.01 and *p <* 0.001, Figure 3E) and total cholesterol (*p <* 0.01 and *p <* 0.001, Figure 3C). In addition, non-HDL-cholesterol plasma concentrations were increased in CTRL compared to chow at two weeks and four months (*p <* 0.05 for both time points, Table 2). LDL-C concentrations in plasma were also transiently higher in the CTRL-fed group compared to the chow-fed group at four months (*p <* 0.05, Figure 3D).

### 3.3. The NASH Diet Increased Liver Weight, Hepatic Triglyceride and Cholesterol Content and Reduced Glycogen Content in Syrian Hamsters

**Figure 4:**
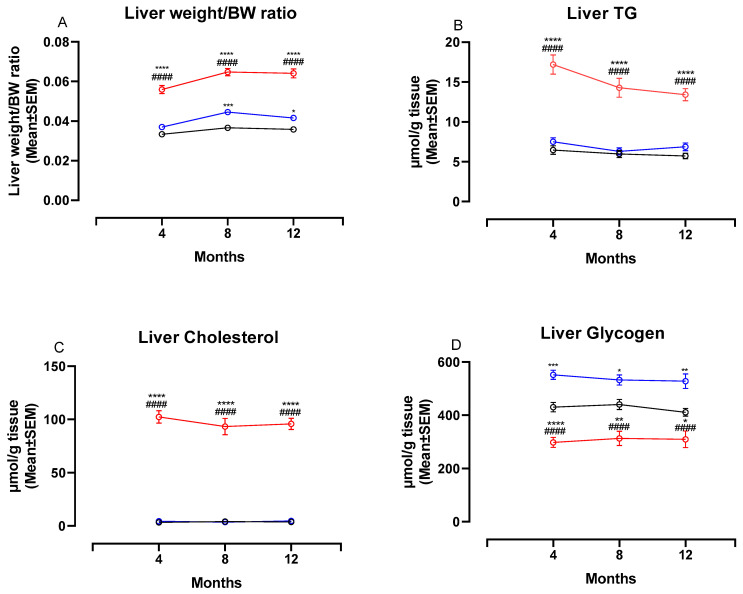
Liver weight ratios and biochemistry in NASH- and control diet-fed Syrian hamsters during the study period. Effects of the NASH-diet on liver weight/BW ratio (**A)**, liver TG (**B**), liver cholesterol (**C**) and liver glycogen (**D**) during the study period. Black circles and lines indicate data from chow-fed hamsters. Blue circles and lines indicate data from CTRL-fed hamsters. Red circles and lines indicate data from NASH-fed hamsters. Superscripts *, **, ***, **** indicate *p* < 0.05, *p* < 0.01, *p* < 0.001, *p* < 0.0001 compared to Chow. Superscript #### indicate *p* < 0.0001 compared to CTRL. BW: Body weight, TG: triglyceride.

Figure 4 shows changes in liver weight/BW ratio and liver biochemistry at four, eight and 12 months. The liver weight:BW ratio was significantly higher in NASH-fed animals compared to both chow- and CTRL-fed animals at four, eight and 12 months (*p <* 0.0001 for all comparisons, Figure 4A). In addition, the two control diets affected the liver differently, with the liver weight:BW ratio being significantly higher in CTRL-fed hamsters compared to chow-fed hamsters at eight and 12 months (*p <* 0.001 and *p <* 0.05, Figure 4A). Biochemical analysis revealed significantly higher hepatic concentrations of both TG and cholesterol in NASH-fed hamsters at all time points (*p <* 0.0001 for all comparisons, Figure 4B,C). Hepatic concentrations of TG and cholesterol did not differ between the two control groups. The hepatic glycogen concentrations were significantly lower in the NASH-fed hamsters compared to both CTRL and chow at four (*p <* 0.0001), eight (*p <* 0.0001 and *p <* 0.01) and 12 months (*p <* 0.0001 and *p <* 0.05, Figure 4D). In addition, chow-fed hamsters had significantly lower glycogen concentrations compared to CTRL at all time points (*p <* 0.001, *p <* 0.05 and *p <* 0.01, Figure 4D).

### 3.4. The NASH Diet Induced Hepatic Steatosis, Inflammation and Fibrosis in Syrian Hamsters

**Figure 5:**
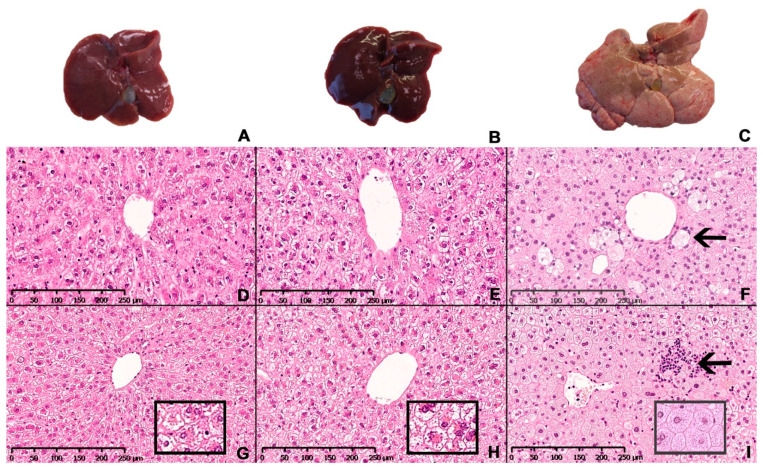
Hepatic macroscopic appearance and histology in Syrian hamsters after exposure to either the NASH diet or the two control diets for eight or 12 months. Macroscopic appearance of excised livers from chow-, CTRL- and NASH-fed hamsters after 12 months (**A**–**C**, respectively). Representative histological sections of liver stained with H&E from each diet group (chow, CTRL, NASH from left to right) at eight (**D**–**F**) and 12 months (**G**–**I**). Inserts in (**G**–**I**): magnified examples (20×) of hepatocyte morphology in chow, CTRL and NASH, respectively, showing pinpoint steatosis in NASH-fed hamsters. Arrow in (**F**): enlarged macrophage in NASH-fed hamsters after eight months. Arrow in (**I**)**:** inflammatory foci containing primarily mononuclear inflammatory cells in liver of NASH-fed hamsters after 12 months.

**Figure 6:**
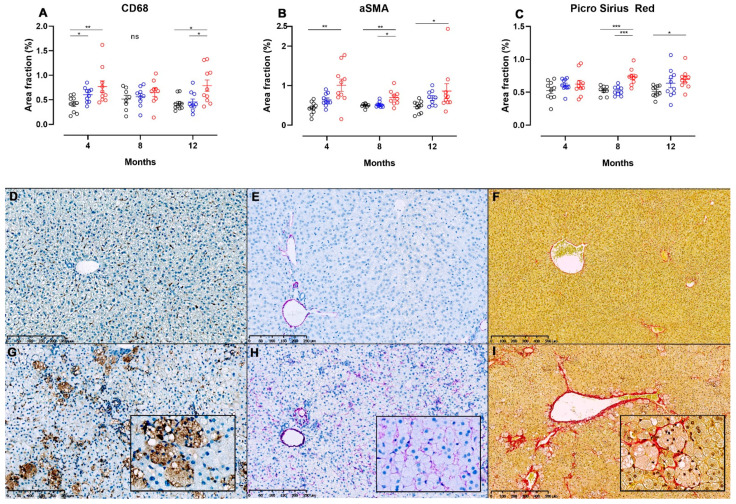
Image analysis of liver sections from NASH- or control diet-fed Syrian hamsters stained for detection of hepatic inflammation, stellate cell activation and fibrosis. Area fractions of CD68+ (**A**) and α-SMA+ (**B**) cells from image analysis of IHC-processed liver sections in chow, CTRL and NASH-fed hamsters at four, eight and 12 months. (**C**) Area fractions of PSR+ staining in chow, CTRL and NASH-fed hamsters at four, eight and 12 months. Black circles indicate chow-fed hamsters, blue circles indicate CTRL-fed hamsters, red circles indicate NASH-fed hamsters. *, **, *** indicate *p* < 0.05, *p* < 0.01, and *p* < 0.001 between groups. (**D**–**I**) Liver sections showing differences in positive staining of cells/fibres in CD68-, αSMA- and PSR-stained sections of liver from chow (middle row, **D**–**F**) and NASH-fed hamsters (**G**–**I**) at 12 months. Inserts in (**G**–**I**) are magnifications (20×) of (**G**–**I**).

Representative images of the macroscopic appearance of the liver and H&E-stained liver sections from chow-, CTRL- and NASH-fed hamsters at eight and 12 months are shown in Figure 5. The four-month time point was not included as histology was similar to eight months. The architecture of the liver parenchyma was visibly altered in the NASH-fed hamsters at eight and 12 months (Figure 5F,I), with enlarged hepatocytes containing pin-point steatosis (Figure 5I, insert), inflammatory foci containing primarily mononuclear inflammatory cells scattered throughout the liver parenchyma (arrow in 5I) and enlarged cells with a partly clear cytoplasm (arrow in 5F). These cells displayed positive staining for CD68 and were therefore macrophages (Figure 6). Inserts in 5G and 5H shows morphology of hepatocytes in chow- and CTRL-fed hamsters, respectively. The steatotic hepatocytes in NASH-fed hamsters were located primarily in zone 1 (surrounding portal veins) and 2, sometimes extending into zone 3 (surrounding central veins). Ballooning hepatocytes were not observed during the histopathological evaluation of the liver sections.

Images displaying pathological changes in liver sections stained with PSR and liver sections used for IHC for CD68 and α-SMA from hamsters fed chow and NASH diets for 12 months are shown on Figure 6, together with results from the image analysis at all time points. Infiltration with CD68+ cells was seen in the NASH-fed hamsters (Figure 6G), and on image analysis CD68+ area fractions were significantly higher in these animals compared to those fed chow diet at four and 12 months (*p <* 0.01 and *p <* 0.05), and those fed CTRL diet at 12 months (*p <* 0.05, Figure 6A), however no significant differences between groups were seen at the eight-month time point. Interestingly, the area fraction of CD68+ cells was also higher in CTRL-fed hamsters compared to chow at four months (*p <* 0.05, Figure 6A). Hepatic stellate cell activation was observed by significantly increased α-SMA area fractions in NASH-fed hamsters at four, eight and 12 months compared to chow (*p <* 0.01, *p <* 0.01 and *p <* 0.05), and at eight months compared to CTRL (*p <* 0.05, Figure 6B). Examples of liver sections stained for α-SMA+ cells from chow and NASH-fed hamsters at 12 months are shown in Figure 6E,H, respectively. Confirming this, collagen deposition visualized on PSR stained sections of liver was significantly higher in NASH-fed hamsters compared to both CTRL and chow-fed hamsters at eight months (*p <* 0.001), and to the chow-fed hamsters at 12 months (*p <* 0.05, Figure 6C). In most NASH-fed animals, collagen deposition was characterized as mild perisinusoidal fibrosis extending from zone 1 (Figure 6I) and sometimes occurring in association with enlarged macrophages (insert in 6I).

### 3.5. The NASH-Diet Increases Circulating Markers of Liver Dysfunction, β-Oxidation and Inflammation in Syrian Hamsters

Plasma concentrations of 3-β-hydroxybutyrate (a marker of β-oxidation activity [19]) were significantly higher in the NASH-fed hamsters compared to CTRL-fed hamsters at two weeks (*p <* 0.01), one month (*p <* 0.0001), four months (*p <* 0.0001) and eight months (*p <* 0.0001), and compared to chow-fed animals at one month (*p <* 0.0001), four months (*p <* 0.05) and eight months (*p <* 0.0001, Table 2). There were no differences in plasma 3-β-hydroxybutyrate concentrations between the CTRL and chow groups (Table 2). Plasma concentrations of ALT were significantly higher in NASH-fed animals compared to CTRL and chow-fed hamsters at one (*p <* 0.01), four (*p <* 0.01 and *p <* 0.05) and eight months (*p* < 0.0001), whereas plasma concentrations of AST were only transiently elevated compared to chow and CTRL at eight months (*p <* 0.05 and *p* < 0.001, Table 2). Circulating plasma concentrations of haptoglobin were significantly elevated in NASH compared to CTRL after two weeks (*p <* 0.01, Table 2), one month (*p <* 0.01), eight months (*p <* 0.001) and 12 months (*p <* 0.001). Haptoglobin concentrations were also slightly elevated in plasma from NASH-fed hamsters compared to chow throughout the study, although the difference only reached statistical significance at two weeks (*p <* 0.01) and eight months (*p <* 0.01, Table 2).

## 4. Discussion

In the present study, we show that Syrian hamsters fed NASH diet for up to 12 months developed marked dyslipidemia and pathological alterations in the liver with resemblance to NAFLD/NASH including hepatic steatosis, infiltration with inflammatory cells, increased activation of hepatic stellate cell, and development of mild fibrosis. Furthermore, while the NASH diet did not induce hyperglycemia or hyperinsulinemia, it did influence the body composition of the hamsters. This is to our knowledge the first study where the effects of a NASH-inducing diet in hamsters were monitored for a year. Of note, this study also indicates a marked effect from the type of control diet, with significant differences between CTRL- and chow-fed hamsters for several endpoints.

In humans, dyslipidemia as well as several other metabolic disturbances (e.g., insulin resistance and obesity) often co-exist with NAFLD/NASH [20,21,22]. The relationship between dyslipidemia and NAFLD is bidirectional, as an increase in circulating plasma lipids can result in ectopic fat deposition in liver tissue, and hepatic fat accumulation may contribute to an atherogenic dyslipidemic profile through e.g., increased very-low density lipoprotein (VLDL)-secretion [23] and possibly by increased CETP-activity and decreased expression of the LDL-receptor [14,24,25]. Increased VLDL-secretion, CETP-activity and decreased expression of the LDL-receptor in the context of diet-induced experimental NAFLD have all been demonstrated previously in hamsters [26,27]. In this study, both plasma concentrations of TG, LDL-cholesterol and HDL-cholesterol were increased in the NASH-fed hamster. While these observations are in agreement with other studies in high fat, high-fructose, high cholesterol diet-induced hamster models of dyslipidemia and hepatic steatosis [26,27,28], there are also studies showing no change in HDL-cholesterol in hamsters fed a diet containing fat, carbohydrates and cholesterol [29,30]. The dyslipidemia observed in human patients with NAFLD is typically characterized by increased plasma TG concentrations, increased or unchanged LDL-cholesterol and decreased HDL-cholesterol [31]. As such, the dyslipidemia of the NASH-fed hamster does not fully mirror the dyslipidemic profile of human patients with NAFLD with not only LDL-cholesterol but also HDL-cholesterol concentrations increasing in the NASH-fed hamsters compared to both control groups. However, although hamsters do carry a higher proportion of their cholesterol in the LDL-fraction compared to mice and rats, it is still a HDL-predominant rodent species [12]. The overall increase in plasma cholesterol in each fraction is likely not only due to the high cholesterol content of the diet, but also the content of other dietary sources such as saturated fats and trans fatty acids as well as total caloric intake [32]. NAFLD in humans is often associated with atherogenic dyslipidemia [33,34,35], where the qualitative nature of LDL-particles is important. Thus, it is not necessarily an increase in total LDL-cholesterol but rather an increase in the fraction of small dense LDL-particles that is indicative of an adverse atherogenic profile and increased risk of cardiovascular disease [36]. While we did not assess the qualitative nature of the LDL-population (with e.g., high-performance gel-filtration chromatography or nuclear magnetic resonance spectroscopy), we did calculate non-HDL-cholesterol concentrations. The non-HDL-cholesterol concentration has been suggested as a non-invasive biomarker for NASH [37,38], and also as a predictor of cardiovascular disease mortality [39,40]. Accordingly, NASH-fed animals displayed higher non-HDL-cholesterol plasma concentrations compared to chow and CTRL at all time points during the study. This agrees well with the significantly worsened liver pathology in this group, as exemplified by hepatic steatosis, inflammation and fibrosis.

Besides dyslipidemia, insulin resistance often coexist with NAFLD and NASH [1,41]. Neither plasma glucose nor RIIE was elevated in NASH-fed hamsters at any time point, and calculations of HOMA-IR indexes did not show significant differences between groups. There were, however, other parameters in the NASH-fed hamsters which could indicate a mildly blunted response to insulin. For example, plasma FFA was significantly increased in NASH hamsters after only two weeks and throughout the study, which could indicate a blunted response of the adipose tissue to the anti-lipolytic effects of insulin [42]. Moreover, significant lower concentrations of hepatic glycogen were observed in the NASH-fed hamsters, which could indicate mild hepatic insulin resistance due to the accumulation of intrahepatic fat [43]. In addition, plasma concentrations of 3-β-hydroxybutyrate were elevated in NASH hamsters. As insulin indirectly inhibits ketogenesis [44], the rise in 3-β-hydroxybutyrate could also be indicative of a blunted insulin response.

Previous studies have shown that diets with high content of fructose, cholesterol and fat in rodent models can induce histopathological changes in the liver resembling NAFLD and features of the metabolic syndrome [45,46,47]. Although NAFLD is not always associated with obesity [48], it is a very common comorbidity in NAFLD patients [1,49,50]. Whether the hamsters on NASH diet in this study should be characterized as obese was entirely dependent on the control diet used for comparison. Compared to CTRL-fed hamsters, NASH-fed hamsters did become obese, however, there were no differences in fat mass compared to chow-fed hamsters, although NASH-fed animals had significantly less lean mass at all time points compared to this group. In agreement with these findings, other studies comparing a high-fat, high-carbohydrate, high-cholesterol diet to undefined chow diets have also shown lack of induction of obesity in hamsters [26,27,30].

In this study, increased area fractions of CD68+ cells in NASH-fed hamsters quantitatively confirmed the presence of inflammation in the liver. CD68 is a surface pan-macrophage marker, and Kupffer cells express this marker on their cell surface [51]. Kupffer cells play an important role in the development and progression of the inflammatory process in NASH [52]. Their activation in NAFLD is mediated by several factors such as free fatty acids, gut-derived endotoxins, signalling molecules from hepatocyte damage as well as cholesterol and its associated metabolites [52]. Both increased plasma concentrations of FFA and increased hepatic and plasma cholesterol concentrations were observed in the NASH-fed hamsters and could therefore have contributed to the increased hepatic CD68 area fractions. The activation of Kupffer cells elicit a secretion of cytokines (e.g., TNF-α, IL-6, IL-1β and CCL2 (MCP-1)) and TGF-β to recruit circulating inflammatory cells and initiate hepatic stellate cell activation, respectively [52]. In agreement with this, hepatic stellate cell activation and subsequently fibrosis was induced in the NASH-fed hamsters.

Ballooning hepatocytes were not observed in hamsters on NASH diet in this study. In humans, ballooning of hepatocytes is an important histopathological hallmark of NASH [53]. Ballooning hepatocytes have been reported in other hamster models of NASH [11,28,54] using a higher dietary cholesterol content than in the present study. Whether the difference in cholesterol content could be the reason for the absence of ballooning in our model is unknown. It should also be noted that presence of ballooning hepatocytes in rodent NASH models is considered somewhat controversial and often has not been validated by IHC [55]. Only one of the above-mentioned studies applied IHC for confirmation of ballooning hepatocytes [11]. Surprisingly, the proposed ballooning cells were found negative for cytokeratin 18 as well as sonic hedgehog. This is in contrast to what has previously been described in humans with NASH, where ballooning cells were found negative for cytokeratin 18 but stained positive for sonic hedgehog [56].

In human NAFLD, the presence of fibrosis is a predictor of overall- and disease-specific mortality [57,58]. As such, the search for drugs which can prevent or reverse fibrogenic processes in the liver of NAFLD patients are a subject of intense research [59], and animal models which develop overt fibrosis or at least initial stages of fibrosis are therefore needed. The NASH-fed hamsters in this study did in fact show increased activation of hepatic stellate cells (indicating initiation of a fibrogenic response) already at four months, and this activation remained throughout the study. PSR area fractions as measures of fibrosis were increased in NASH hamsters compared to both control diets at the eight-month time point. At the 12-month time point, a significant difference was only observed between chow- and NASH-fed hamsters. However, the average PSR area fraction for CTRL-fed hamsters were fully comparable at eight and 12 months, but somewhat more variable at the 12-month timepoint. This larger variation explains why the PSR area fraction was not significantly different between CTRL- and NASH-fed hamsters at 12 months. The relative increase in PSR area fraction in NASH-fed hamsters compared to control diets was somewhat modest at eight and 12 months (30–35%) and the hamsters only developed mild perisinusoidal fibrosis. It is possible that exposure to the NASH-diet for a longer period than 12 months would result in development of more prominent fibrosis (i.e., bridging fibrosis), however, studies of such duration are generally not desirable for efficient testing of potential drug candidates.

Recent studies in hamster models of NAFLD/NASH have reported that it is possible to induce most of the hallmarks of NASH in hamsters fed diets high in fat, with some combination of added carbohydrate and/or cholesterol during a shorter time span (12–25 weeks) [26,28,29,30,54,60]. In the study done by Briand and colleagues, hamsters were fed a free-choice diet (choice between chow and a high-fat diet (40.8 kcal% fat, 44.4 %kcal carbohydrate and 0.5% cholesterol) with 10% fructose in drinking water) for 12–25 weeks. Histological evaluation of H&E- and PSR-stained sections revealed presence of hepatocyte ballooning, inflammatory foci and bridging fibrosis as early as 12 weeks, accompanied by biochemically quantified hepatic steatosis and increased plasma ALT concentrations. While aggravated histopathology was induced earlier in the study by Briand et al. compared to this study (e.g., bridging fibrosis), measures of inflammation, hepatocyte damage and fibrosis were not formally quantified until after 25 weeks. In another recent study, hamsters were fed either a chow diet or a high-fat, high-cholesterol diet (supplementation of chow diet with 11.5 % corn oil, 11.5% coconut oil and 1% cholesterol) for 12 weeks. This led to hepatic steatosis, inflammation, stellate cell activation and bridging fibrosis, as well as increased hepatic expression of cytokines and profibrogenic markers [30] already after 12 weeks. In addition, the study by Lai et al. compared the effects of the high-fat, high-cholesterol diet in C57BL/6 mice to that of the hamsters, and data indicated that hamsters were more prone to diet-induced detrimental histopathological changes in the liver [30]. The discrepancies in terms of time to development of lesions between the present study and those described above could be due to the relatively higher cholesterol content of the diet in previous studies (0.5–1% in previous studies vs 0.3% used in the present study). Moreover, there is some indication that using a free-choice diet—as opposed to a one-diet regimen—impacts consumption patterns and thereby exposure to the detrimental components of experimental diets [28,61]. Lastly, the difference in diet composition as well as the use of liquid fructose vs. addition of fructose to the diet [28] could play a role. It has been suggested that sugar administered as liquids may be more effective in inducing metabolic syndrome compared to the solid form [62,63].

As described above, there are several points where the findings in this hamster model differ from human NASH, i.e., the hamsters did not become hyperinsulinemic or hyperglycaemic, ballooning hepatocytes were not observed and HDL-cholesterol increased in hamsters fed the NASH-diet. While the hamster is therefore not a perfect model, it is still a model where a plasma lipid profile with some resemblance to human dyslipidemia and several important histological features of NAFLD can be induced using diets with lower concentrations of cholesterol compared to the cholesterol concentrations typically seen in NASH-inducing diets used in mouse and rat models. It has been suggested that concentrations of dietary cholesterol for hamsters should not exceed 5–10 times that of the normal intake in humans, which in hamsters would be accomplished by cholesterol concentrations between 0.1–0.3% [64], as used in the present study. In a previous study in rats, the NASH diet with 2% cholesterol did not induce dyslipidemia after 16 weeks of feeding [47], while the hamsters in the present study developed dyslipidemia already after two weeks on a NASH diet with 0.3% cholesterol. Additionally, it has been reported that hamsters have higher concentrations of hepatic cholesterol than rats when feeding diets with comparable cholesterol content [65]. Furthermore, when comparing mice and hamsters fed the same high-fat, high cholesterol diet, hamsters developed more severe liver pathology and had higher ALT/AST plasma concentrations, compared to mice [30]. For exploration of the interplay between NAFLD and dyslipidemia, which frequently occurs simultaneously in patients [1], the hamster model described in this study therefore has higher translational value than mouse and rat models.

Other limitations are that corrections of analyses for multiple testing across endpoints were not performed in this study and that the study included only male hamsters. Due to the use of only male hamsters, findings are not translational to the general human population. Effects of the NASH diet in female hamsters should have been included in order to fully evaluate the translational potential of the model. This limitation should be kept in mind, when interpreting the results of the present study.

Another interesting finding in this study is the impact of choice of control diet in interpretation of the metabolic and histopathological outputs of the model. Compared to CTRL-fed hamsters, NASH hamsters became dyslipidemic, obese and developed hepatic steatosis. However, for several of the comparisons of inflammatory and fibrosis-related histopathological parameters there was no significant difference between CTRL-fed and NASH-fed hamsters. When the chow diet was used for comparison, NASH-fed hamsters became dyslipidemic but not obese. However, in addition to the development of hepatic steatosis, more comparisons of inflammation- and fibrosis-related endpoints reached statistical significance when NASH-fed hamsters were compared to chow-fed hamsters. It has been suggested that the use of semi-purified diets as opposed to diets typically termed “chow”-diets, presents some advantages for use in experimental studies [66,67]. These include identifiable one-source nutrients, open-label formulas, minimal batch-to-batch variation and protection from non-nutrient sources of variation (e.g., phytoestrogens and heavy metals) [68,69]. The present study does not allow us to decipher which differences between the CTRL and chow diet that led to the phenotype variability in some of the readouts in this study but it supports the notion that choice of control diet may have an important impact on the study outcome and interpretation.

Finally, the metabolizable energy of the three diets used in the study was not similar (chow: 3.3 kcal/g; CTRL: 3.7 kcal/g; and NASH: 4.3 kcal/g). As we did not measure food intake, we are not able to conclude how these differences in energy densities might have influenced the results. Food intake was not measured due to the length of the study, the large number of animals and the inherent difficulties associated with measuring food intake in this species (restraining of hamsters several times a week to empty cheek pouches for remaining food pellets, and removal of pellets distributed on the cage floor). These limitations should be considered when interpreting the results of this study.

## 5. Conclusions

In the present study, we demonstrate that hamsters fed a high-fat, high-fructose, high-cholesterol diet develop prominent dyslipidemia characterized by increased plasma FFA, TG, total cholesterol, LDL-C and HDL-C after only two weeks. Moreover, within four to eight months, the NASH-diet induced several pathological alterations in the liver which are also seen in human NAFLD, i.e., hepatic steatosis, inflammation, stellate cell activation and fibrosis.

## Figures and Tables

**Table 1:** Composition of the chow, CTRL and NASH diets used in the study *.

Macronutrient Contribution	Altromin 1324 (Chow)	D16010104 (CTRL)	D16010102 (NASH)
Fat (kcal%)	11	10	40
Protein (kcal%)	24	20	20
Carbohydrates (kcal%)	65	70	40
Total	100	100	100
Metabolizable energy (kcal/g)	~3.3	3.7	4.3

* A more detailed diet composition can be viewed in Supplemental Table S1.

**Table 2:** Plasma parameters in chow, CTRL, and NASH-fed hamsters during the study.

	Sampling Time Point														
*Plasma parameters*	2 Weeks			1 Month			4 Months			8 Months			12 Months		
	Chow (Mean ± SD)	CTRL (Mean ± SD)	NASH (Mean ± SD)	Chow (Mean ± SD)	CTRL (Mean ± SD)	NASH (Mean ± SD)	Chow (Mean ± SD)	CTRL (Mean ± SD)	NASH (Mean ± SD)	Chow (Mean ± SD)	CTRL (Mean ± SD)	NASH (Mean ± SD)	Chow (Mean ± SD)	CTRL (Mean ± SD)	NASH (Mean ± SD)
Glucose ^†^ (mmol/L)	3.0 ± 0.4	3.0 ± 0.7	3.0 ± 0.7	3.0 ± 0.3	3.1 ± 0.5	3.4 ± 0.7	4.4 ±0.6	4.9 ± 1.6	4.0 ± 0.8	3.9 ± 0.7	4.1 ± 0.6	4.1 ± 0.5	5.0 ± 0.6	6.1 ± 1.3 *	**5.0 ± 0.7 ^#^**
Insulin ^†^ (RIIE)	-	-	-	-	-	-	183.4 ± 62.5	205.9 ± 127.7	194.5 ± 120.3	201.4 ± 136.7	249.1 ± 159.6	248.9 ± 195.7	245.6 ± 79.6	258.8 ± 89.2	296.9 ± 242.8
HOMA-IR index ^†^	-	-	-	-	-	-	5.51 ± 2.55	8.31 ± 5.09	7.83 ± 6.55	6.53 ± 3.59	6.17 ± 4.29	8.70 ± 7.95	9.09 ± 3.40	11.52 ± 3.94	11.15 ± 9.63
3-β-hydroxybutyrate ^†^ (µmol/L)	88.8 ± 29.7	67.8 ± 26.8	**117.3 ± 41.1 ^##^**	84 ± 22.8	73.8 ± 26.0	174.6 ± 40.9 ****^,####^	227.5 ± 75.8	180.1 ± 44.0	304.9 ± 109.2 *^,####^	161.6 ± 49.75	148.1 ± 43.6	273.5 ± 62.7 ****^,####^	172.2 ± 42.7	162.6 ± 61.7	265.4 ± 135.8
Haptoglobin ^†^ (g/L)	0.3 ± 0.1	0.3 ± 0.1	0.5 ± 0.2 **^##^	0.5 ± 0.1	0.4 ± 0.2	**0.6 ± 0.1 ^##^**	0.8 ± 0.2	0.7 ± 0.3	0.9 ± 0.2	0.6 ± 0.2	0.5 ± 0.2	0.8 ± 0.2 **^###^	0.7 ± 0.3	0.5 ± 0.2	**1.0 ± 0.4 ^###^**
ALT ^†^ (U/L)	32.3 ± 5.3	33.1 ± 13.1	36.1 ± 0.5	41.0 ± 10.8	39.7 ± 7.7	59.0 ± 16.6 **^,##^	75.6 ± 68.5	63.1 ± 26.8	114.2 ± 72.3 *^,##^	48.1 ± 13.8	43.5 ± 14.4	86.8 ± 34.1 ****^,####^	79.5 ± 22.0	82.0 ± 29.1	101.8 ± 39.0
AST ^†^ (U/L)	27.6 ± 11.7	48.9 ± 34.1	32.3 ± 15.8	27.7 ± 5.6	32.3 ± 8.7	46.8 ± 44.9	45.5 ± 22.0	43.4 ± 14.6	56.0 ± 35.3	27.2 ± 5.5	25.0 ± 3.5	31.7 ± 6.8 *^,###^	37.8 ± 5.7	47.6 ± 14.0	41.1 ± 11.8
Non-HDL-C ^†^ (mmol/L)	0.8 ± 0.2	1.0 ± 0.2 *	1.8 ± 0.3 ****^,####^	1.2 ± 0.2	1.3 ± 0.3	2.3 ± 0.4 ****^,####^	1.1 ± 0.2	1.5 ± 0.3 *	3.2 ± 0.6 ****^,####^	0.7 ± 0.2	0.9 ± 0.3	2.8 ± 1.1 ****^,####^	0.8 ± 0.2	0.8 ± 0.3	3.1 ± 1.3 ****^,####^

All results are given as mean ± SD (standard deviation). Results in bold indicate where significant differences exists between the effects of the diets. *, **, and **** indicate significant differences compared to chow at *p* < 0.05, *p <* 0.01, and *p <* 0.0001, respectively. ^#^, ^##^, ^###^ and ^####^ indicate significant differences compared to CTRL at *p <* 0.05, *p <* 0.01, *p <* 0.001 and *p <* 0.0001, respectively. Parameters marked with the superscript ^†^ were transformed by the natural logarithm before being analysed. RIIE: Rat insulin immunoactivity equivalents; ALT: Alanine aminotransferase; AST: Aspartate aminotransferase; HDL-C: High-density lipoprotein cholesterol; HOMA-IR: Homeostatic model assessment of insulin resistance.

## Data Availability

All data presented in this study are available on request from the corresponding author. The data are not uploaded in publicly accessible databases due to corporate rules from Novo Nordisk A/S.

## Supplementary Materials

The following are available online at https://www.mdpi.com/2072-6643/13/2/604/s1, title, Table S1: Detailed composition of the chow, CTRL and NASH diets, Table S2: Immunohistochemistry (IHC) protocols for staining of liver sections.
