# Supplementary material for: Temporal Development of Dyslipidemia and Nonalcoholic Fatty Liver Disease (NAFLD) in Syrian Hamsters Fed a High-Fat, High-Fructose, High-Cholesterol Diet
Source: Nutrients. 2021 Feb 12;13(2):604. doi: 10.3390/nu13020604 (PMC7917647; doi:10.3390/nu13020604)
Supplement: Supplementary file 1 [file nutrients-13-00604-s001.zip › Supplemental table S1. Detailed diet composition.docx]

| **Macronutrient contribution** | **Altromin 1324 (chow)*** | |  | **D16010104 (CTRL)** | | **D16010102 (NASH)** | |
| --- | --- | --- | --- | --- | --- | --- | --- |
| Fat (kcal%) | 11 | |  | 10 | | 40 | |
| Protein (kcal%) | 24 | |  | 20 | | 20 | |
| Carbohydrates (kcal%) | 65 | |  | 70 | | 40 | |
| Total | 100 | |  | 100 | | 100 | |
|  |  | |  |  | |  | |
| **Ingredient** | gm | kcal | **Ingredient** | gm | kcal | gm | kcal |
| Casein | - |  | Casein | 200 | 800 | 200 | 800 |
| Cystine | 3.2 |  | L-Cystine | 3 | 12 | 3 | 12 |
|  |  |  |  |  |  |  |  |
| Corn starch | - |  | Corn starch | 550 | 2200 | 0 | 0 |
| Maltodextrin 10 | - |  | Maltodextrin 10 | 150 | 600 | 100 | 400 |
| Fructose | - |  | Fructose | 0 | 0 | 200 | 800 |
| Glucose | - |  | Glucose | 0 | 0 | 0 | 0 |
| Sucrose | - |  | Sucrose | 0 | 0 | 96 | 384 |
| Disaccharides | 47.8 |  |  |  |  |  |  |
| Polysaccharides | 391.2 |  |  |  |  |  |  |
|  |  |  |  |  |  |  |  |
| Cellulose | - |  | Cellulose | 100 | 0 | 100 | 0 |
|  |  |  |  |  |  |  |  |
| Soybean Oil | - |  | Soybean Oil | 25 | 225 | 25 | 225 |
| Corn Oil, Partially Hydrogenated | - |  | Corn Oil, Partially Hydrogenated | 0 | 0 | 135 | 1215 |
| Lard | - |  | Lard | 20 | 180 | 20 | 180 |
|  |  |  |  |  |  |  |  |
| Mineral Mix S10026 | - |  | Mineral Mix S10026 | 10 | 0 | 10 | 0 |
| Phosphorus | 5.0 |  | DiCalcium Phosphate | 13 | 0 | 13 | 0 |
| Calcium | 7.0 |  | Calcium Carbonate | 5.5 | 0 | 5.5 | 0 |
| Potassium | 8.8 |  | Potassium Citrate | 16.5 | 0 | 16.5 | 0 |
|  |  |  |  |  |  |  |  |
| Vitamin Mix V10001 |  |  | Vitamin Mix V10001 | 10 | 40 | 10 | 40 |
| Choline Chloride | 1.1 |  | Choline Bitartrate | 2 | 0 | 2 | 0 |
|  |  |  |  |  |  |  |  |
| Cholesterol | - |  | Cholesterol | 0 | 0 | 2.8** | 0 |
|  |  |  |  |  |  |  |  |
| FD&C Blue Dye #1 | - |  | FD&C Blue Dye #1 | 0.05 | 0 | 0 | 0 |
| FD&C Red Dye #40 | - |  | FD&C Red Dye #40 | 0 | 0 | 0.05 | 0 |
| Total | 1000 | ~3277 | Total | 1106.6 | 4057 | 938.85 | 4056 |

**Supplemental Table S1.** Detailed composition of the chow, CTRL and NASH diets

* The detailed energy content in kcal of diet constituents was only partially available from the provider of the Altromin 1324 diet (Brogaarden, Denmark).

** Corresponding to ~0.3% Cholesterol
