# Supplementary material for: Temporal Development of Dyslipidemia and Nonalcoholic Fatty Liver Disease (NAFLD) in Syrian Hamsters Fed a High-Fat, High-Fructose, High-Cholesterol Diet
Source: Nutrients. 2021 Feb 12;13(2):604. doi: 10.3390/nu13020604 (PMC7917647; doi:10.3390/nu13020604)
Supplement: Supplementary file 1 [file nutrients-13-00604-s001.zip › Supplemental table S2. Immunohistochemistry protocols.docx]

**Supplemental table S2.** Immunohistochemistry (IHC) protocols for staining of liver sections

|  | **IHC protocol** | |
| --- | --- | --- |
|  | **CD68** | **α-SMA** |
| *Antibody* | Rabbit anti-CD68 | Rabbit anti-αSMA |
| *Catalog number and provider* | Ab125212 (Abcam) | Ab5694 (Abcam) |
| *Clonality and isotype* | Polyclonal (IgG) | Polyclonal (IgG) |
| *Dilution* | 1:100 (5 µg/ml) | 1:333 (0.6 µg/ml) |
| *Blocking of unspecific protein binding* | Discovery inhibitor (Roche) 12 min, TNB buffer (Roche) 20 min | Discovery inhibitor (Roche) 12 min, TNB buffer (Roche) 20 min |
| *Antigen retrieval* | CC1 buffer (Roche) (pH 8.4), 95°C, 24 min | CC1 buffer (Roche) (pH 8.4), 95°C, 24 min |
| *Wash buffer* | Reaction buffer (Roche) | Reaction buffer (Roche) |
| *Detection* | BrightVision Goat anti-Rabbit (37 °C, 30 min) | BrightVision anti-Rabbit (37 °C, 20 min) |

Abbreviations: CC1: cell conditioning 1; IHC: Immunohistochemistry; CD68: cluster of differentiation 68; TNB: Tris-NaCl buffer; α-SMA: alpha smooth muscle actin.
